# Barriers to and facilitators of effective communication in perinatal care: a qualitative study of the experiences of birthing people with sensory, intellectual, and/or developmental disabilities

**DOI:** 10.1186/s12884-022-04691-2

**Published:** 2022-04-26

**Authors:** Gul Saeed, Hilary K. Brown, Yona Lunsky, Kate Welsh, Laurie Proulx, Susan Havercamp, Lesley A. Tarasoff

**Affiliations:** 1 Dalla Lana School of Public Health, University of Toronto, 155 College Street, Toronto, Ontario M5T 3M7 Canada; 2 Department of Health and Society, University of Toronto Scarborough, 1265 Military Trail, Toronto, Ontario M1C 1A4 Canada; 3 Azrieli Adult Neurodevelopmental Centre, Centre for Addiction and Mental Health, 250 College Street, Toronto, Ontario M5T 1R8 Canada; 4 Nisonger Center, The Ohio State University, McCampbell Hall, 1581 Dodd Drive, Columbus, OH 43210-1257 USA

**Keywords:** Accessibility, Blindness, communication, Developmental disabilities, Deafness, perinatal care, Pregnancy, Qualitative research, Sensory disabilities

## Abstract

**Background:**

Effective provider-patient communication is a key element of quality health care, including perinatal care. What constitutes “effective communication” in perinatal care may vary according to the population seeking care, such as women with intellectual and developmental disabilities (IDD) and sensory disabilities. Research broadly indicates that communication issues are among the barriers to perinatal care experienced by women with disabilities. However, few studies have explicitly explored their communication experiences in this context. The purpose of this study was to understand the communication experiences of birthing people with IDD and/or sensory disabilities in perinatal care.

**Methods:**

We conducted semi-structured interviews with 17 people with IDD (e.g., autism, cognitive delay) and/or sensory disabilities (e.g., d/Deaf, blind) in Ontario, Canada, who had recently given birth, to explore barriers to and facilitators of effective communication in perinatal care. A combination of deductive and inductive thematic analysis guided data analysis.

**Results:**

We found that birthing people with IDD and/or sensory disabilities encountered multiple barriers to effective communication in perinatal care, namely, lack of policies and guidelines, lack of provider experience, lack of provider effort, as well as ableism and provider assumptions. Facilitators included knowledgeable, aware, and supportive providers; access to communication aids and services; tailoring information to patients’ disability-related communication needs; empathic communication; and, communication among providers.

**Conclusion:**

Unmet communication needs may contribute to negative health and social outcomes for birthing people with disabilities and their newborns. Accessibility policy implementation and practice change are needed to meet the communication needs of people with IDD and/or sensory disabilities in perinatal care to ensure positive experiences and outcomes.

## Background

As a key component of patient-centered care [1], effective communication is critical for providing high-quality and safe care [2, 3], leading to better outcomes for patients, including improved health, fewer adverse events, and shorter hospital stays [4]. Communication in health care is effective when there is a bidirectional flow of information between health care providers and patients [5]. All parties must be able to understand and interpret the information shared [3], including a patient’s medical history, diagnosis, treatment plan, and concerns [2], to effectively communicate and, in turn, engage in shared decision-making [6, 7]. Good communication is particularly vital for achieving the ethical and legal process of informed consent as it ensures patients have sufficient and relevant information, and are able to comprehend information so they can independently make informed decisions about their own medical care [7, 8]. Information in health care settings is typically disseminated orally (e.g., in conversations), in writing (e.g., procedural instructions), or electronically (e.g., emails) [9]. Communication can be enhanced by paying attention to non-verbal cues, including body language, eye gaze, and facial expressions [5, 10]. Effective communication may be compromised when individuals who have a limited capacity to use the typical modes of health care communication are not provided with alternative methods due to factors such as providers’ lack of competency, putting them at risk for misdiagnosis and mistreatment [11].

Poor communication is an important determinant of preventable adverse maternal and neonatal health outcomes [12]. Yet, poor communication remains widespread in perinatal care [13]. This led the World Health Organization (WHO) to develop a framework to improve the quality of perinatal health care, which includes eight domains of quality maternity care, including effective communication, defined as “communication with women and their families [that] … responds to their needs and preferences.” [14] The literature suggests effective communication is clear, direct, and includes informed, shared decision-making [14–16]. Empathic communication, in particular, leads to effective information exchange and reduces patients’ anxieties [17]. Effective communication also encourages women to access perinatal care, while poor communication is associated with reluctance to access care, which may increase risks for adverse outcomes [5].

What facilitates effective communication may vary according to the population seeking care, such as women with disabilities. The literature on perinatal care experiences of women with disabilities generally [17–20] and of those with intellectual and developmental disabilities (IDD) [21, 22] and sensory disabilities specifically [23, 24] indicates these groups report poor communication as a barrier to care. A U.K. survey found only 66% of women with IDD felt health care providers spoke to them in a way they could understand compared to 84% of women without disabilities [18]. Qualitative studies from the U.S. and Poland found health care providers lacked awareness of the disability-related needs of pregnant women with hearing and vision loss, which caused providers to avoid direct communication or ignore their needs [23, 24]. In addition to challenges directly related to communication, pregnant and pregnancy-planning women with IDD and sensory disabilities face attitudinal barriers, including health care providers questioning their parenting abilities [25, 26]. These barriers undermine the reproductive autonomy of women with disabilities and hamper their ability to effectively engage with providers. These barriers may also have significant implications for women with disabilities, who are at elevated risk for adverse perinatal outcomes, including preeclampsia, caesarean delivery, and preterm birth [27–29]. While studies have identified communication issues as one of several perinatal care barriers encountered by women with IDD and sensory disabilities, few studies have explicitly examined their communication experiences.

Guidelines have established maternity care standards to increase access for women, including those with disabilities [14, 30]. The U.K.’s Framework for Maternity Services Standards states maternity services must consider communication needs of women with disabilities [30]. Several countries have implemented legislation to protect the right to barrier-free health care for people with disabilities. The Americans with Disabilities Act forbids discrimination against people with disabilities in all spheres of public life, including health care, and requires health care providers to give reasonable modifications to make services accessible [31]. In Ontario, Canada, the Accessibility for Ontarians with Disabilities Act strives to reduce barriers, including communication barriers, that prevent persons with disabilities from participating in society [32]. While progress has been made to address differential access to health care among persons with disabilities, perinatal care standards for women with disabilities remain neglected. Given that pregnancy rates in women with disabilities are rising [33], this topic warrants attention.

As such, the purpose of this study was to understand the communication experiences of women with IDD and/or sensory disabilities in Ontario, Canada, in the context of perinatal care.

## Methods

This study is part of a larger project exploring the perinatal care experiences of birthing people with physical disabilities, sensory disabilities, and IDD who had recently given birth in Ontario, Canada. The present study used a qualitative design to understand the communication-related perinatal care experiences of the participants with IDD (e.g., autism, cognitive delay) and/or sensory disabilities (e.g., d/Deaf, blind) (*N* = 17). We use the language of “birthing people”, as not all participants identified as cisgender women. Ethics approval was obtained from the Research Ethics Board at the University of Toronto (reference # 35018); all methods were performed in accordance with relevant guidelines and regulations from the Declarations of Helsinki and the Tri-Council Policy Statement on Research Ethics.

### Sampling and recruitment

We employed convenience sampling in combination with purposeful sampling [34] to recruit individuals with disabilities who were diverse in terms of socio-demographic characteristics such as geography and race/ethnicity. Our goal was to include a minimum of 10 people with sensory disabilities and 10 with IDD. Study flyers were distributed via electronic listservs, newsletters, and websites of over 100 organizations and groups that serve people with disabilities, parents, and pregnant/postpartum people in Ontario. We also recruited participants through our team’s networks and the study Advisory Committee, which is comprised of ~ 30 individuals, including people with disabilities, clinicians, community organization staff, and provincial policy representatives, who provide strategic and knowledge translation inputs to the research team. To reach d/Deaf participants, we worked with a Deaf community organization to create a recruitment video using American Sign Language (ASL). Individuals interested in participating contacted the study coordinator and were screened by telephone to determine eligibility.

Individuals who met the following inclusion criteria were eligible: (a) self-identified as having a sensory disability and/or IDD, (b) resided in Ontario, (c) were 18 years of age or older, (d) had a livebirth in the last 5 years, and (e) were able to converse in English or ASL. To assess capacity to consent, individuals with IDD were asked additional screening questions [35, 36].

### Data collection

Interviews were conducted in-person (*n* = 11) or virtually (e.g., telephone and Zoom, *n* = 6), based on the needs and preferences of participants, and were conducted by a public health researcher and peer researchers (two women with physical disabilities) from July 2019 to February 2020. Before each interview, participants provided written informed consent and completed a questionnaire about their socio-demographics (e.g., age, relationship status, education level), disability (e.g., type), and pregnancy history (e.g., number of children birthed).

Interviews followed a guide that was developed in collaboration with the study Advisory Committee and consisted of questions and probes following the chronology of participants’ most recent perinatal care experience, beginning with the time before pregnancy, followed by initiation of prenatal care, care during pregnancy, labour and delivery, and early postpartum care. Interviews were conducted individually, with the exception of one participant who chose to have a language interpreter as English was their second language, three d/Deaf participants who required ASL interpreters, and five participants who desired to have a support person (e.g., parent, caseworker) present. Support persons signed a confidentiality agreement before the interview. With one exception, their responses are not included herein. Interviews were audio-recorded with the participants’ consent and averaged 69 min (range: 36–120 min).

Upon interview completion, participants received a CAD 50 gift card (e.g., grocery store) and a list of resources on mental health, social services, and violence against women. The lead researcher connected with each participant within 1 week post-interview to ask how they were doing and if there was anything else they might like to add regarding their perinatal care experience. No participants finished the interview early, withdrew from the study, or reported difficulties post-interview.

### Data analysis

The interviews were transcribed intelligent verbatim (i.e., omission of pauses, filler words, and utterances) by a professional transcription service and a student volunteer, and subsequently verified for accuracy and de-identified by the interviewers.

For the purpose of this study, we focused our analysis on participants’ responses about communication in the context of perinatal care. We used a combination of deductive and inductive thematic analysis [37] to identify common and disability-specific themes across the dataset. A deductive approach to thematic analysis, which aims to fit data into pre-existing codes based on existing literature, guided development of our initial coding framework [37, 38]. Meanwhile, inductive thematic analysis, a data-driven approach, allowed us to focus on emerging insights from participants’ experiences and add nuance and specificity to the coding framework [38].

The first and last author developed an initial coding framework based on domains of effective communication identified in the literature deemed to be relevant to perinatal care broadly and to birthing people with IDD (e.g., provider used non-technical terms) and sensory disabilities (e.g., access to ASL interpreters) specifically. They independently line-by-line coded three interview transcripts (one representing each disability type: IDD, hearing loss, vision loss), to highlight words or phrases related to the codes in the initial framework, and to derive new codes that captured concepts pertaining to communication-related perinatal care experiences. They then discussed their coding, resolved differences, and refined the coding framework (analyst triangulation [39]). The coding framework was further revised after discussion of one interview transcript with the larger author team. All interviews were coded according to the refined coding framework using NVivo 12 software by the first author. Throughout the analysis process, the coding framework was modified as new codes were added when data did not fit into existing codes. Coded data were used to construct theme memos; similar codes were organized and grouped into potential themes and subthemes. Several iterations of the theme memos were discussed and revised by the first and last author before sharing with the second author for further discussion. Ultimately, an overview of the findings was shared with the study Advisory Committee and a draft of the findings was shared with the larger author team.

## Results

### Participant characteristics

Seven participants identified as having a sensory disability only, six as having an IDD only, and four as having multiple disabilities (IDD and a physical or sensory disability). Two participants were under age 20, nine were between the ages of 26 and 34, and six were age 35 or older. Many participants (*n* = 11) were married or in a committed relationship. Most (*n* = 14) identified as heterosexual and one identified as genderqueer/non-binary. Four participants were born outside Canada and twelve identified as white. Most (*n* = 13) resided in large urban centers (population 150,000 to 2.8 million), and four in medium and small towns (population 1800 to 75,000). Levels of education varied, with participants with IDD reporting lower education levels (*n* = 9, high school or less); five participants with a sensory disability reported having a college diploma (*n* = 2), undergraduate degree (*n* = 2), or graduate degree (*n* = 1). Details about participants’ disabilities and birth histories are provided in Table 1. All participant names are pseudonyms.

**Table 1:** Participant disability and birth history details

Pseudonym	Disability (self-reported identity or diagnosis)	Assistive device, communication aid, and/or service use	Number of children birthed^a^	Type and place of birth (most recent)	Primary perinatal care provider(s)^b^ (most recent)
Ava	Sensory (Deaf)	ASL interpreters	4	Cesarean, hospital	Obstetrician
Corey	IDD (autistic) + Physical (Ehlers-Danlos syndrome)	Cane, walker, wheelchair	1	Vaginal, hospital	Obstetrician
Crystal	IDD (cognitive delay)	–	4	Vaginal, hospital	Midwife
Danielle	IDD (chromosomal abnormality)	–	2	Vaginal, hospital	Obstetrician
Emilie	Sensory (hearing loss in one ear)	–	2	Vaginal, home	Midwife
Garima	IDD	–	3	Vaginal, hospital	Midwife, obstetrician
Heidi	IDD	–	4	Cesarean, hospital	Obstetrician
Jasmine	IDD (fetal alcohol spectrum disorder [FASD])	Assistive communication technologies (Scribe, Kurzweil)	1	Vaginal, hospital	Obstetrician
Laura	IDD (chromosomal abnormality) + Physical (scoliosis)	–	2	Vaginal, hospital	Nurse practitioner, obstetrician
Leah	IDD (mild intellectual disability) + Sensory (blind in one eye)	–	4	Vaginal, hospital	Obstetrician
Mackenzie	Sensory (Deaf)	ASL interpreters	3	Cesarean, hospital	Midwife, obstetrician
Maria	IDD (multiple mild intellectual disabilities)	–	3	Vaginal, hospital	Obstetrician
Robyn	Sensory (Deaf, Usher syndrome)	Cane (in crowds), ASL interpreters, video relay service	3	Cesarean, hospital	Obstetrician
Shannon	Sensory (Deaf)	Hearing aids, ASL interpreters	1	Vaginal, hospital	Nurse practitioner, family physician
Siobhan	Sensory (blind)	White cane, screen-reader	4	Vaginal, home	Midwife
Tala	Sensory (severe hearing loss) + IDD (developmental disability)	Hearing aids	1	Vaginal, hospital	Obstetrician
Zahra	Sensory (blind)	White cane, assistive communication technologies	1	Vaginal, hospital	Obstetrician

^a^Two participants’ children have since died (1 was stillborn; 1 died within a week of being born)

^b^Some participants had more than one primary perinatal care provider or shared care due to where they lived (e.g., rural or small town) and/or the nature of their pregnancy (e.g., if complications arose, if they had twins)

### Themes

We categorized findings under two overarching themes: barriers to and facilitators of effective communication in primary perinatal care and community care (see Table 2). We define “primary perinatal care” as the standard perinatal care most birthing people access, often in hospital settings, including appointments with health care providers (e.g., obstetrician, midwife), ultrasounds, labour and delivery care, and early postpartum care. We define “community care” as optional services, such as prenatal education classes, provided in home and community settings by different types of providers (e.g., public health nurse, lactation consultant). (In Ontario, primary care and community care delivered by public health are delivered free of charge.)

**Table 2:** Summary of Barriers to and Facilitators of Effective Communication

Theme	Definition
**Barriers to Effective Communication**	
Lack of Policies and Guidelines	Lack of institutional policies and guidelines on communication accommodations/access in primary perinatal care and community care settings and in turn failure to provide communication accommodations and recognize barrier-free communication as a right
Lack of Provider Experience	Providers’ lack of experience working with and knowledge about people with disabilities and their communication needs
Lack of Provider Effort	Providers’ lack of effort to tailor or adapt information (or the delivery of information) to patients’ disability-related communication needs
Ableism and Provider Assumptions	Providers’ negative assumptions about patients’ disabilities and their communication needs
**Facilitators of Effective Communication**	
Knowledgeable, Aware, and Supportive Providers	Providers who were knowledgeable, aware, and supportive concerning patients’ disabilities and their communication needs
Access to Communication Aids and Services	Provision or accessibility of ASL interpreters, braille resources, and alerting devices (e.g., auditory devices for blind/low vision persons and vibrating devices for d/Deaf persons)
Information Tailored to Patients’ Disability-related Communication Needs	Providers who tailored information (or the delivery of information) to meet patients’ disability-related communication needs
Empathic Communication	Providers who carefully listened to and understood patients’ needs and accommodated disability-related needs without fixating on them
Communication among Providers	Communication among health and social service providers about patients’ disability-related communication needs

### Barriers to effective communication

#### Lack of policies and guidelines

Across all groups, participants shared that there appeared to be a lack of policies and guidelines regarding communication accommodations in perinatal care, especially in hospital settings. For instance, Robyn noted they found “no real standards” on booking interpreters at hospitals. Consequently:

“They don’t have interpreters. … [or] sometimes, the hospitals will have their own interpreter so they’re not calling up the interpreter from [qualified interpreting agency] … Usually, it’s cost-benefit to the hospitals but not necessarily a benefit to me because they are not exactly as well-versed or as well-trained as [qualified interpreting agency] interpreters.”

Participants who were d/Deaf shared they frequently lacked access to qualified ASL interpreters because hospitals refused to book them, indicating that they are costly, and leaving patients to pay for interpreters themselves. This barrier was exacerbated for those who lived in smaller cities due to the dearth of qualified interpreters. For instance, Shannon, who lived in a remote Northern Ontario community, described their labour and delivery care experience:

“When we went to the hospital, I was saying that we need to have an interpreter for the delivery, the actual birth. They [hospital administration] were very resistant because they would have to spend [money]; they would have to pay a lot of money to find someone [interpreter] to stay in the community for a month [because] they didn’t know how long and when the baby would come.”

Similarly, several participants shared that prenatal education classes and public health and parenting programs did not provide access to ASL interpreters, making these important community-based services inaccessible to them. Robyn, who birthed twins, recounted:

“I phoned them and asked if they would be willing to hire an interpreter and they said, ‘No, you’re more than welcome to come as long as you pay for the interpreter yourself.’ And they got a program specifically for people who are pregnant with twins but unfortunately, because they refused to provide an interpreter, there was no way I could participate.”

Although the onus was often on the patient to advocate for their communication needs, participants who were d/Deaf, including Shannon, reported: “There was nothing specific about being Deaf, you know, tips on how to get an interpreter or stuff like that. There’s nothing.”

As well, few community care services tailored information to participants’ disability-related communication needs. In some instances, participants shared that they relied on their peers and vice-versa for additional support, including how to provide care for their newborn:

“I helped a friend figure out how to feel the clothes and know like this is 0 to 3 months and this is 3 to 6 months. And how to make a system and organize all these baby clothes … how to burp and bath the baby … With my other blind friends who are parents, I helped teach them a lot of those things.” (Siobhan)

Moreover, participants reported that barrier-free communication was not recognized as a right in perinatal care. Shannon noted:

“There’s nothing out there. There’s no like, ‘This is your right to have access.’ Even if you go to any hospital now, you’d see that if you want an interpreter, then you can ask for it. But in the birth wing they don’t say, ‘You need an interpreter. This is your right to have access.’”

In the absence of policies and guidelines regarding communication access, participants shared that having their communication needs met was largely dependent on the individual service providers they interacted with; “a lot of the time, like, accommodations comes from the person [provider] being thoughtful on their own” rather than a standard practice, noted Siobhan.

#### Lack of provider experience

Participants shared that their communication needs were frequently unmet due to providers’ lack of experience working with and knowledge about people with disabilities and their communication needs. Siobhan described the challenges they faced while accessing services to manage gestational diabetes:

“My first time at the diabetes clinic … the standard thing was, ‘Okay, go downstairs and get yourself a meter and go home and test’ … But I don’t know how to find the pharmacy downstairs, I don’t know how to get a glucose meter once I’m there. Once I get the glucose meter, I don’t know how to read the instructions, I don’t know how to test … I’ve never done this before. … I was asking for assistance too and they [medical staff] were just like ‘Uhh.’”

Participants also encountered providers who did not understand the utility or specificity of communication aids, including alerting devices. Robyn described their situation during a hospital stay:

“I said, ‘I could bring my technical devices from home but you’re going to hate those devices because they’re going to be ringing, the lights are going to be going off and everything else.’ And she [the nurse] said, ‘Well, I’ve got an idea.’ She goes ‘Here’s the heart monitor that you can put on. You watch this heart monitor and if the light flashes on this heart monitor, it means baby’s awake.’ So I said, ‘The light flashing that means he’s crying or what does it mean?’ She goes, ‘No. Well, if his heart stops, that means the light will go off.’ And I said, ‘But how am I going to know if he’s crying?’”

Due to providers’ lack of knowledge and experience concerning disability, several participants felt the onus was on them to educate providers about disability and their communication needs in addition to having to self-advocate for their communication needs. Jasmine, who had fetal alcohol spectrum disorder (FASD), shared such an experience, which their mother, whom they requested to have present at the interview, further explained: “very often … we had to educate them [providers]” about Jasmine’s disability and needs.

#### Lack of provider effort

Participants described that their communication needs were often unmet in perinatal care settings because providers made no effort to tailor information to their disability-related communication needs. For instance, Siobhan, whose third child died due to a congenital heart defect, shared their ultrasound experience during their fourth pregnancy:

“I’ve had many ultrasounds over the four pregnancies and that was actually the only time where I had someone totally silent the whole time. And that was also when I really wanted to know what was going on … When she [ultrasound technician] was through I said to my husband, ‘Is there a baby there?’ … ‘Do you see arms, legs, the head? … Is there two babies?’ like I have no idea, right? Whereas a sighted person can see the screen and okay, they can’t interpret what they’re seeing but they can at least say, ‘Hey look! There’s a moving baby on the screen’ … ”

Zahra, who was blind, shared a similar experience regarding a public health nurse and home visitor, who made no effort to adapt parenting information to their needs:

“I remember they used to tell me … ‘We are playing a [DVD] to the parents so that they can know how to play with their babies, but for you we cannot play a [DVD] because you can’t see it.’ So that means that they don’t have other options, so they [need] more methods [of] teaching in accordance with the people.”

Providers failed to tailor written communication, such as pamphlets, to the communication needs of blind participants:

“When they give information to parents, they give you a bunch of pamphlets, right? … ‘Here’s a bunch of mysterious papers,’ you know? And usually [I] just have to recycle it because I don’t know what this is so I do really appreciate anytime someone can email me that, as a PDF or anything. Or even just tell me the key [information], so it is helpful … So just making that information a bit more accessible is helpful.” (Siobhan)

Participants with sensory disabilities shared that providers refused to provide access to communication aids and services. Several participants who were d/Deaf, like Mackenzie, reported that all types of providers involved in their care “refused to contact an interpreter”:

“The pediatrician, she refused to get interpreters, and she would get mad with us because we were screwing up the formula measurements. … [son] had to go to [Neonatal Intensive Care Unit, NICU] for a few weeks … and the NICU refused to bring in an interpreter. … I was with [the public health program] and [they also said] ‘no, I don’t want to pay for an interpreter, I don’t want to get an interpreter.’”

Robyn described how, when they experienced a complication around 20 weeks and went to the emergency department, the provider was dishonest about having booked an ASL interpreter:

“I asked for an interpreter … they said, ‘Oh yeah, we’ve already called them.’ And that’s a very common thing with deaf people, whether you get to the hospital, you say you need an interpreter, they say, ‘Oh yeah, we’ve already called them,’ and then they never show up because they never actually got the call. … Later I called [qualified interpreting agency] to find out that they never even actually got a call for an interpreter.”

Rather than providing communication access, participants described that providers frequently relied on their family members to facilitate communication: “They [providers] would have my husband interpret for me, but it’s still not full information …” (Mackenzie); “They would ask my daughter to interpret for me, you know? She’s 3 and a half!” (Robyn).

#### Ableism and provider assumptions

Participants reported several instances of providers making assumptions about their disability-related communication needs, which prevented access to communication aids and services. In Shannon’s case, providers did not have knowledge about the hearing spectrum, specifically that some people with hearing loss can read lips and talk but may still require interpreters to assist with communication:

“When I go to the doctors, if I start talking, they think I can hear everything and they start talking, and then they turn their heads and I miss what they’re saying. And it could be something that’s important and they said it but I didn’t get it because I didn’t hear it. And they tend to forget that so it becomes a barrier, because especially when it’s something medical, there’s like, ‘Well, I don’t understand you. I need an interpreter.’ And they don’t understand why I need an interpreter because I can speak so well. Just because I can speak well doesn’t mean I can understand what you’re saying.”

For most participants with IDD, providers typically were not aware of their disability and, in turn, their communication needs because of how their disabilities presented. Although disclosure of disability status was important for having their communication needs met, participants with IDD frequently reported that (fear of) involvement with child protection services and previous negative encounters with providers, who seemingly did not know a lot about (their) disability, made it difficult to do so. For instance, Danielle, who had chromosomal abnormality, explained:

“I didn’t even tell my own doctor [about my disability]. … [They] probably wouldn’t understand it anyways so I don’t bother. … People don’t understand it. … People think that they know what it is but I don’t know, they don’t.”

Similarly, Crystal shared they did not disclose their disability because they did not “want to be treated different, labelled different, because of my disability. I want people to think of me as just [Crystal], you know? Not as, ‘Oh, there’s [participant] with a disability.’”

### Facilitators of effective communication

Although not as frequently reported by participants as barriers, we also discerned facilitators of effective communication at the provider level.

#### Knowledgeable, aware, and supportive providers

Knowledgeable, aware, and supportive perinatal care providers played a central role in fulfilling patients’ communication needs. Shannon, who was Deaf but could speak and read lips, described how their family doctor understood that their communication needs were unmet because they had no interpreter present during an ultrasound that included screening for Down syndrome:

“He actually had to sit down … with my husband and myself for an hour afterward, just going over and explaining the reasons why we had to do the test and what he thought and that there’s nothing. He went like, ‘Wow, I have to do this because you didn’t have the information, you didn’t hear it properly.’ … I was lucky when the doctor jumped in and said, ‘No, she needs it,’ and that’s probably how we got the interpreter.”

The communication needs of patients with sensory disabilities were more likely to be met if their provider had previous experience working with people with sensory disabilities:

“I don’t know if [hospital] was being lazy and didn’t want to [get an interpreter], but that’s when the doctor ordered them to say you have to do this, it’s her right, and its twins, so like there’s going to be several appointments. So, they reluctantly did that, but that doctor had the knowledge of what was expected where [as] other doctors may not have had the same. I think that doctor had said he had in the past delivered for other deaf parents, so he had the prior experience.” (Mackenzie)

Across all groups, participants indicated that supportive providers respected their communication access requests and tailored information accordingly. Robyn described how their provider fulfilled their request:

“I made sure that the doctor understood—I actually got him to sign something to say that he would actually put the baby up over the curtain because my peripheral vision, if I’m not looking straight up … I was going to be missing that baby come over the sheet after the C-section. So I did not want to miss that so the doctor was like, ‘Okay, I’ll do that.’”

Similarly, Heidi, who had IDD, shared that after they disclosed their disability to their doctor, he checked in to make sure they understood what was communicated to them: “He just tried to talk to me. He would ask, ‘Do you understand?’, and I would go, ‘Yeah’, and then he’s like, ‘Okay, so it’s mostly just spelling or doing essays parts … ’”.

Participants noted that providers who were aware and supportive of their communication needs were more likely to make time to describe and explain things in detail. Participants who were blind and those with IDD shared that when providers did this, they were able to better understand the information communicated to them, increasing access to information. Leah, who had a mild intellectual disability and was blind in one eye, commented: “They [providers] explain but they should sit down and actually explain a little better, more, you know.” Tala, who had a developmental disability and severe hearing loss, shared that when their prenatal education class instructor became aware that they were facing communication challenges, the instructor took the time to fully explain class material, which helped them understand it better.

#### Access to communication aids and services

For participants with sensory disabilities, access to communication aids (e.g., hearing aids, braille resources) and services (e.g., interpreting services) was a key element of effective communication in perinatal care. Participants who were d/Deaf reported that access to qualified ASL interpreters was crucial in perinatal care, with Ava, who was Deaf, commenting: “to provide interpreters, that should be a must.” Importantly, participants articulated that interpreters were also essential for the providers to communicate with their patients:

“Get an interpreter from the get go, because it’s for them; the interpreter’s for them [the provider], not for me. … If a baby has a heart problem, they bring in a cardiologist to focus on that problem, so the interpreter’s the same thing. … They think the interpreter’s just for me, that they’re paying out of pocket. I’m like no, it benefits you. ... They really need to have an interpreter more like their staff member.” (Mackenzie)

Given the intimate nature of childbirth, some participants remarked: “In a perfect world, I would have preferred if we picked the interpreter and we would have picked a more qualified interpreter … someone who I trust as opposed to a stranger.” (Shannon).

Participants emphasized the importance of having interpreters present throughout the perinatal period and especially around labour and delivery due to the unpredictability of this period. For example, Mackenzie recounted that their providers sent the interpreter home after a few hours in the hospital and had to call them back “10 minutes later” as they were rushed in for a cesarean delivery but “by the time the interpreter arrived the babies have been delivered.”

As well, participants noted that having access to an ASL interpreter throughout the postpartum hospital stay was important:

“They’re [providers] like, ‘Okay, we know you need an interpreter, you need it for the delivery.’ Because that’s a big part of giving birth but then they forget about the after part. There’s a lot of continuing discussion of care. What if your child has health issues and you want to discuss them about how to feed them? Do they need specific formula? … There’s so many different things that could potentially happen and you need interpreters for that.” (Shannon)

Participants who were blind identified a need for braille resources and tactile modes of communication. For instance, Siobhan, whose third child was diagnosed with a heart defect during pregnancy, shared:

“They take time to teach you something about the heart and how the heart works so you learn about the normal heart and then [they] show the parents what is different about their child’s heart, right? And the model they had of the heart can be tactile so I love that I could feel the 4 atriums and the arteries and the veins and it was all tactile. And the doctor took the time to show me like, ‘Okay feel here, this is the pulmonary artery and this is the one where there's usually a valve here but your daughter doesn’t have it,’ then I could feel all of those things. So I really appreciated that there was a tactile model.”

#### Information tailored to patients’ disability-related communication needs

Participants indicated that providers tailoring relevant information to their disability-related communication needs was essential. Across all groups, especially for those with IDD, this consisted of providers using non-technical terms. For instance, when asked about what recommendations they would make to perinatal care providers to improve care for birthing people with disabilities, Garima stated: “Stop talking doctor terms … not everyone’s going to understand what hemorrhaging is.”

Participants who were blind and those with IDD, such as Jasmine, who had “difficulty processing information,” shared that when providers explained things step-by-step and included hands-on demonstrations, they were better able to understand instructions and learn how to do tasks. Siobhan described their experience receiving a hands-on demonstration:

“My CNIB [Canadian National Institute for the Blind] independent skills worker … actually walked [me] through how to change a diaper. … It does help to have someone really take the time to show you how to do it, not just like, ‘Watch this video on how to change a diaper,’ you know? But more like, ‘Feel this, this is the diaper. This is how you can feel the flaps, this is the back, this is the front.’... That was good.”

Providers tailoring information to the communication needs of participants who were d/Deaf or had hearing loss included a combination of ASL, written communication, and gestures, with the latter two being especially critical when an ASL interpreter was not present. For example, Robyn, who was Deaf and had Usher Syndrome, shared: “I told them [obstetrician] specifically how to communicate with me, tapping me on the arm, letting me know that I need to be attentive to a decision or something like that.”

Some participants with IDD, including Crystal, preferred written communication: “With the cognitive delay, sometimes it’s hard for me to learn. ... But if it’s written for me, I can do it.”

#### Empathic communication

Related to the aforementioned facilitators, participants emphasized that providers listening carefully and understanding their needs and perspectives was crucial for effective communication. For instance, Corey, who was autistic and had a physical disability, shared: “The most positive support I got was from people who would actually listen to what I either wanted or how I felt about stuff or how I was feeling...”

Providers making an effort to fully engage in conversation with participants was deemed an important part of empathic communication, wherein participants felt comfortable sharing their needs and concerns. As an example, Crystal shared:

“She [midwife] was awesome. … She turned off every computer in her office, she didn’t write anything down … she was like, ‘Tell me anything you want to tell me, even if it’s about Children Aid, just let it loose,’ you know? And I was able to talk to her and I didn’t feel like she was writing anything down at the time. So I didn’t have to stress that, ‘Oh, no! What is she writing?’ kind of thing. It made me feel more at ease, to know that she was actually sitting there, listening to me.”

Providers who accommodated disability-related communication needs without getting “stuck” on those needs and treating patients differently because of their disability also facilitated effective communication, as they helped patients feel more comfortable with the provider. Corey shared:

“The ones who were a lot more just treating me like a regular person in the sense of like, you know, not assuming that I can’t do something or – instead of treating me more like a child. Because that’s something I find a lot in general with medical professionals is they can treat you that way, especially with autism. … just treat people as people and not as the diagnosis.”

While participants appreciated when providers met their communication needs, they wished that providers would see beyond their disability and focus on their overall experience and identity as a new parent. Siobhan shared that after they experienced the loss of their third child, a provider seemed more preoccupied with the fact that they had braille grief resources to share rather than offering comfort:

“I’m thinking about my dying baby, I’m like, ‘This is the last moment … my blindness is the last thing on my mind,’ you know? And someone being all excited to get to accommodate my blindness … Like I wished she just humbly, quietly passed me the resources … like she would have to a sighted person and she would have done something like, ‘Hey, we’re so sorry that you’re going through this. Here’s some resources if you need them.’”

#### Communication among providers

For most participants, perinatal care encompassed more than primary perinatal care and included interactions with multiple providers in various settings (e.g., fertility service providers, prenatal educators, pediatricians). Therefore, providers communicating their patients’ disability-related communication needs to one another was vital to facilitate access to communication aids and services throughout the perinatal period. For example, Mackenzie explained that their pediatrician contacted child protection services (Children’s Aid Society [CAS] in Ontario) but failed to mention that they were Deaf:

“That’s when the CAS nightmare really began … the doctor did not mention to CAS that I was deaf, she failed to say that. So, the CAS Emergency Response showed up and said ‘I want in, I want to see,’ and I was floored because I had no communication; there was no interpreter, there was nothing.”

## Discussion

From our analysis of the communication-related perinatal care experiences of birthing people with IDD and/or sensory disabilities, we identified four barriers to and five facilitators of effective communication in the context of perinatal care. Barriers were lack of policies and guidelines, lack of provider experience, lack of provider effort, as well as ableism and provider assumptions. Facilitators included what was done and by whom to meet patients’ communication needs, including knowledgeable, aware, and supportive providers; access to communication aids and services; tailoring information to patients’ disability-related communication needs; empathic communication; and, communication among providers. Participants with sensory disabilities and IDD shared common overall barriers and facilitators; however, the ways in which some of these manifested depended on disability type, for example, in the specific types of communication accommodations required. Although several participants shared positive communication-related experiences, most reported experiencing numerous barriers to effective communication that warrant attention by perinatal care providers and decision-makers.

Consistent with prior studies on the perinatal care experiences of women with IDD [22] and sensory disabilities [24], our study shows that effective communication in perinatal care is vital. Similar to Hubbard et al.’s study on d/Deaf women in the U.S., we found that access to ASL interpreters was critical for effective communication between providers and d/Deaf patients [23]. We also identified other factors, including the quality of and familiarity with the interpreter, that must be considered when providing access to ASL interpreters. For those with IDD, Malouf et al.’s U.K.-based study found that midwives explaining information in non-technical terms helped women understand information [22]. While consistent with our findings, our inter- (IDD vs. sensory disabilities) and intra- (blind vs. d/Deaf) group comparisons revealed that providers using simple language facilitated clear communication across all groups, and especially for birthing people with IDD. Further, our finding that birthing people with IDD and sensory disabilities frequently face communication barriers in perinatal care is consistent with previous research. For example, providers’ lack of knowledge and awareness about people with disabilities and their needs have been identified as major communication barriers in perinatal care for women with IDD in the U.K. [22] and women with vision loss in Poland [24]. Our study adds to this literature by showing that birthing people with IDD and sensory disabilities share similar experiences when they access perinatal care within the Canadian health care system. We also show that communication barriers extend beyond primary prenatal care (e.g., appointments with obstetricians, hospital visits), to the postpartum period, with several participants reporting their communication needs were unmet in community care, including public health programs and in appointments with pediatricians.

Given the Canada Health Act’s tenants of universality and accessibility [40], which all provinces must follow, it was surprising that many participants reported they did not have access to communication aids and services, including ASL interpreters, in hospitals in Ontario. Our findings highlighted the absence of policies and guidelines on communication in perinatal care to support providers in making these services available; thus, whether patients’ communication needs were met depended greatly on individual providers. To our knowledge, there is no requirement for effective communication in perinatal care for birthing people with disabilities in Canada. Lack of support from their institutions may explain why providers made little effort to provide communication access. Providers in Ontario may not be incentivized to spend extra time to fulfill patients’ communication needs because they are often remunerated only for visits of a particular length (e.g., 10–15 min) [41]. Providers’ lack of knowledge and awareness about patients’ disability-related communication needs and their legal duty to accommodate those needs is likely due to insufficient focus on disability in their education and training [22, 42].

This study has some limitations. While our sample included people with multiple disabilities, we could not tease apart how multiple disability-related needs together affected communication. There is a need to explore the unique communication experiences of birthing people with multiple disabilities in perinatal care. Data were collected using in-person and virtual methods, according to the needs and preferences of participants; we did not assess whether the results differed depending on data collection method. Our study did not explore all types of communication needs (e.g., intervenor services for deafblind people), nor did we did we have data to examine all settings (e.g., perinatal mental health care settings). The needs of birthing people with specific disabilities, and in specific perinatal care settings, is an important area for future research. Nonetheless, a strength of our study was the diverse sample in terms of socio-demographics, including geography and education level. Our inclusion of birthing people with IDD and with sensory disabilities also allowed us to make inter−/intra-group comparisons that may be helpful for providers to accommodate the common and unique disability-related communication needs of a broad range of patients with disabilities.

Women with IDD and sensory disabilities have elevated risk of perinatal complications [27]. Similar to prior studies [5, 22], our findings suggest unmet communication needs may contribute to misunderstandings, which could lead to adverse health and social outcomes for birthing people with disabilities and their newborns. For instance, we found a lack of ASL interpreters resulted in participants not accessing services that may have been helpful to them during the prenatal and postpartum periods. Poor communication also places unnecessary stress on families navigating the transition to parenthood and possibly managing perinatal complications. Conversely, participants identified that continuous effective communication helped them manage their own and their newborn’s health perinatally. Communication has been consistently deemed a crucial element of perinatal care in international policy frameworks to improve maternal and neonatal outcomes, including the Lancet framework for quality maternal and newborn care [43] and the aforementioned WHO framework [14]. This study makes a compelling case for the need to meet the communication needs of birthing people with disabilities in perinatal care.

As identified in our study, providers and institutions can make communication accessible by providing qualified ASL interpreters to d/Deaf persons and electronic documents and tactile modes of communication to blind people, and using non-technical terms to explain concepts to those with IDD. One participant suggested that the limited access to qualified ASL interpreters in small communities could be addressed by leveraging technology and implementing virtual interpreting services (e.g., video relay interpreters [VRI]). While VRI may not be feasible in all cases (e.g., during labour), this suggestion shows the need for creative solutions and adaptations in resource-constrained settings to ensure the needs of women with disabilities are met.

To minimize institutional-level barriers to effective communication, there is a need to implement communication policies and guidelines in perinatal care settings to ensure all staff (e.g., perinatal care providers, receptionists) meet standards of effective communication. For example, the Canadian Consensus Guidelines for Primary Care of Adults with IDD recommends that providers implement effective communication [44] and provides concrete strategies. In the U.S., Havercamp et al. (2021) developed a national consensus on disability competencies for medical students, including communication competencies (e.g., “demonstrate communication strategies to best meet the needs/abilities of the patient”) [42]. Example medical education curricula have also been recently published [45, 46]. Such guidelines and curricula can be used to ensure adequate education and training for perinatal care providers to increase their knowledge and awareness about people with disabilities and their communication needs. Given that ableism and provider assumptions were reported by several participants in our study, guidelines and training pertaining to disability are critical for improving provider awareness and attitudes. Further, as many have rightly advocated [47, 48], disability training should be developed and taught in collaboration with people with disabilities to ensure their needs and perspectives are accurately represented.

## Conclusion

Birthing people with IDD and/or sensory disabilities encounter multiple communication barriers in perinatal care that may have health implications for them and their newborns. While participants identified critical facilitators of effective communication, they more frequently reported barriers. Initiatives at the institutional and provider levels, including accessibility policy implementation, provider education, and practices tailored to patients’ disability-related communication needs, are needed to improve communication in perinatal care for birthing people with IDD and sensory disabilities.

## Data Availability

The data analysis is based on individual interviews that due to ethical approval are only available to researchers involved in this study.

## Abbreviations

ASL: American Sign Language
CAS: Children’s Aid Society
CNIB: Canadian National Institute for the Blind
FASD: Fetal Alcohol Spectrum Disorder
IDD: Intellectual and/or Developmental Disabilities
NICU: Neonatal Intensive Care Unit
VRI: Video Relay Interpreters VRI
WHO: World Health Organization
